# Adult Mosquito and Butterfly Exposure to Permethrin and Relative Risk Following ULV Sprays from a Truck-Mounted Sprayer

**DOI:** 10.1007/s00244-023-01022-0

**Published:** 2023-12-07

**Authors:** Timothy A. Bargar, Yongxing Jiang

**Affiliations:** 1 U.S. Geological Survey, Wetland and Aquatic Research Center, 7920 NW 71st Street, Gainesville, FL 32653 USA; 2 City of Gainesville Mosquito Control, 405 NW 39th Ave, Gainesville, FL 32609 USA; 3 Present Address: Indian River Mosquito Control District, 5655 41st Street, Vero Beach, FL 32967 USA

## Abstract

Ground applications of adulticides via a specialized truck-mounted sprayer are one of the most common practices for control of flying adult mosquitoes. Aerosols released to drift through a targeted area persist in the air column to contact and kill flying mosquitoes, but may also drift into adjacent areas not targeted by the applications where it may affect nontarget insects such as imperiled butterflies. This study compared the risk of permethrin to adult mosquitoes and adult butterflies to assess the likelihood that the butterflies would be affected following such sprays. Permethrin toxicity values were determined for *Aedes aegypti* and *Culex quinquefasciatus* (LD50s of 81.1 and 166.3 ng/g dw, respectively) and then combined with published toxicity data in a species sensitivity distribution for comparison with published permethrin toxicity data for adult butterflies. The sensitivity distributions indicated adult butterflies and mosquitoes are similarly sensitive, meaning relative risk would be a function of exposure. Exposure of adult butterflies and adult mosquitoes to permethrin was measured following their exposure to ULV sprays in an open field. Average permethrin concentrations on adult mosquitoes (912–38,061 ng/g dw) were typically an order of magnitude greater than on adult butterflies (110–11,004 ng/g dw) following each spray, indicating lower risk for butterflies relative to mosquitoes. Despite lower estimated risk, 100% mortality of adult butterflies occurred following some of the sprays. Additional studies could help understand exposure and risk for butterflies in densely vegetated habitats typical near areas treated by ULV sprays.

Adult mosquito control is one of the common practices in south Florida where elevated mosquito populations increase the likelihood of disease transmission. In the Florida Keys, permethrin is one of the two insecticides used for the control of flying mosquitoes. It is applied by ultra-low-volume (ULV) sprays after sunset from trucks driving through residential neighborhoods, some of which are adjacent to habitats within either state or federal lands. Ultra-low-volume sprays are considered space sprays (Barber et al. 2007; Bonds 2012) that are intended to drift into residential areas where there are actively flying adult mosquitoes. Given that the spray is intended to drift, it may enter and contaminate nontarget habitats adjacent to the residential areas. Such contamination of nontarget habitats was reported by Pierce (2011) and Bargar (2022). Pierce (2011) reported permethrin contamination of broad-leaved plants in Pine Rockland and hammock habitats, while Bargar (2022) reported permethrin contamination of sawgrass in wetland habitats, both within the National Key Deer Wildlife Refuge in the Florida Keys. Two federally listed butterfly species exist in those habitats. Bartram's scrub hairstreak butterfly (*Strymon acis bartrami* Comstock & Huntington) is a federally threatened species that exists within the pine scrubland habitat while the Palatka skipper (*Euphyes pilatka klotsi* Miller, Harvey, & Miller), which is being considered for federal listing, is a butterfly that depends on the sawgrass wetlands. Other federally listed butterfly species (Miami blue butterfly—*Cyclargus thomasi bethunebakeri* Comstock & Huntington and the Schaus swallowtail butterfly—*Heraclides aristodemus ponceanus* Schaus) exist in nontarget habitats adjacent to residential areas elsewhere in the Florida Keys. Permethrin is categorized by the US Environmental Protection Agency as highly toxic to honeybees and beneficial insects based on standard acute contact and oral toxicity studies leading them to conclude permethrin may impact beneficial insect populations (USEPA 2007). The intersection of contamination potential, toxicity to insects and the presence of imperiled butterflies raises concerns about the possible risk of permethrin to those butterflies.

The coexistence of adult mosquito populations and imperiled butterflies in habitats exposed to mosquito control pesticides means both may be exposed and at risk from that exposure. A risk comparison for butterflies and mosquitoes requires data on toxicity and exposure for both insect groups. Several studies have reported 24-h LD50s for adult mosquitoes exposed to permethrin (Pridgeon et al. 2008, Agramonte et al. 2017, Chansang et al. 2018, Estep et al. 2018, Taylor-Wells et al. 2018, Tak et al. 2020 and Baker et al. 2023). The toxicity values reported in those studies ranged from 49 to 6960 ng/g for five different species (*Culex quinquefasciatus* Say, *Anopheles quadrimaculatus* Say, *Aedes aegypti* Linnaeus, *Anopheles gambiae* Giles and *Aedes albopictus* Skuse). Fewer studies have reported 24-h LD50s for adult butterflies exposed to permethrin (Salvato 2001; Hoang et al. 2011). Those studies reported 24-h LD50 values ranging from 0.4 to 1600 ng/g for five different butterfly species (*Eumaeus atala* Poey, *Heliconius charithonia* Linnaeus, *Junonia coenia* Hubner, *Vanessa cardui* Linnaeus and *Anartia jatrophae* Linnaeus). No studies were found that reported exposure levels of either insect group in the field to permethrin following ULV sprays not allowing a comparison of exposures to the reported toxicities (i.e., risk evaluation). The available toxicity data indicate similar sensitivity of adult butterflies and adult mosquitoes to permethrin, but a lack of exposure data means risk estimations for either group of insects would be highly uncertain. It is critical to determine exposure concentrations for both insect groups following ULV sprays to evaluate whether adult mosquito control may occur with minimal impact to nontarget butterflies in habitats intentionally or unintentionally exposed to ULV sprays.

We conducted a series of experiments to begin generating data that would enable a risk comparison for the two insect groups. Since permethrin toxicity data reported in the literature for mosquitoes were based on nominal exposures, we conducted 24-h acute contact toxicity studies with adult mosquitoes to determine their susceptibility to permethrin and calculate LD50 values based on residues measured on the mosquitoes following the toxicity tests. In addition, permethrin residues on adult mosquitoes and adult butterflies were measured following their exposure to ULV sprays from a truck-mounted sprayer to estimate relative exposure following sprays in the field. Finally, we estimated relative risk for adult butterflies and mosquitoes based on the measured exposures and the toxicity values we determined for mosquitoes and reported in the literature for adult mosquitoes and butterflies. The relative risk will indicate the likelihood of effect to butterflies in habitats contaminated following ULV sprays containing permethrin for adult mosquito control.

## Methods

### 24-h Acute Contact Toxicity Studies

Three different adult mosquito species (*Ae. aegypti, Cx. quinquefasciatus and An. quadrimaculatus*) were purchased from a commercial vendor (Benzon Research Inc, Carlisle, PA). Mosquito age when shipped from the vendor ranged between 6 and 10 days. Upon receipt, all mosquitoes were transferred into and maintained within screened holding cages (each species was held in a separate cage) in a room in which temperature and relative humidity ranged from 23.9 to 26.7 °C and 60 to 85%, respectively. The photoperiod in the room was set to a 16 h:8 h light:dark cycle. Food for the mosquitoes in each cage was available from a cotton wick placed into a cup containing 10% sugar water.

**Fig. 1:**
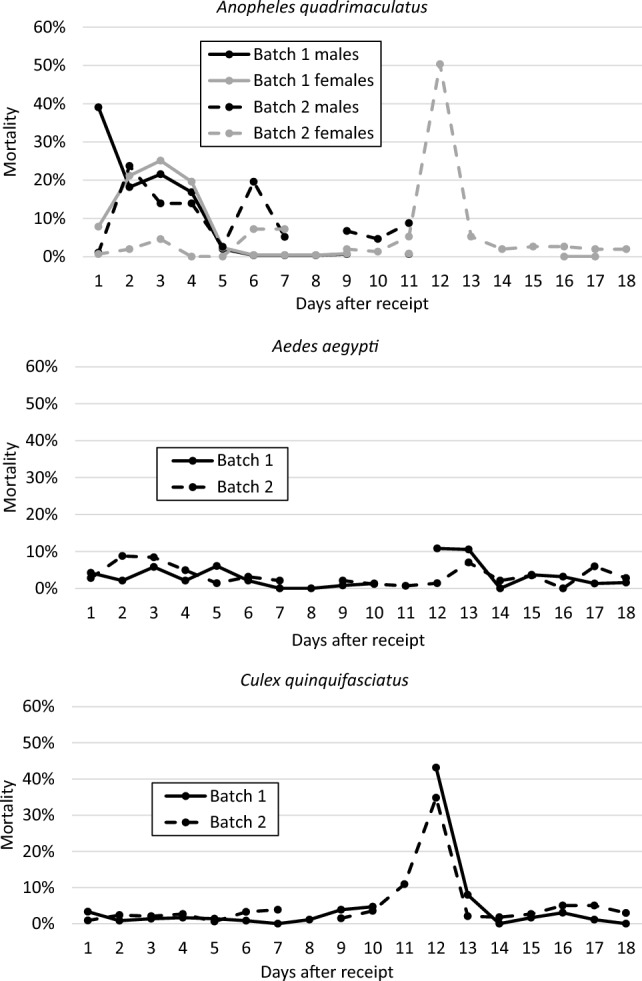
Percent mortality for three different mosquito species in holding cages following receipt from a commercial vendor

Mosquito husbandry was evaluated prior to the toxicity tests to ensure their viability. Husbandry was evaluated by monitoring mosquito survival during the 2–3 weeks after receipt from the vendor. Husbandry conditions were considered acceptable if mortality was less than 10% during that period. Mortality was < 10% up to 10 days after receipt for *Ae. aegypti* and *Cx. quinquefasciatus*, but was > 10% for *An. quadrimaculatus* (Fig. 1). Acute toxicity tests were conducted only on *Ae. aegypti* (hereafter referred to as Aa) and *Cx. quinquefasciatus* (hereafter referred to as Cq) within 1 week of receipt from the vendor.

Toxicity studies with the mosquitoes generally followed the procedures outlined in Pridgeon et al. (2008) and are briefly described below. Mosquitoes were exposed to permethrin (Sigma-Aldrich-certified reference material) dissolved in acetone rather than a pesticide formulation to ensure response is attributable to the active ingredient alone. Range finding tests (24-h exposures) were conducted to determine dose levels for the definitive tests. Three separate definitive tests were run for each species. Each definitive test was comprised of 5 dose levels along with a solvent control (mosquitoes dosed with acetone only) and control. Sample size for each treatment group ranged from 9 to 20 mosquitoes. Each replicate batch of mosquitoes was aspirated from the holding cage into a vial, immobilized by placing the vial into a freezer for 1 min and then poured onto a tissue paper covered frozen cold plate. Using a Hamilton 700 series syringe fitted with a PB 600 repeating dispenser, 200 nL of the respective dose solution or pesticide-free carrier solvent (solvent control) was dispensed onto the thorax of each mosquito within 1 min of being placed onto the cold plate. All dosed mosquitoes were then poured into a cup and secured within the cup by covering it with netting secured in place with a rubber band. The tissue paper was replaced between each replicate batch of mosquitoes to minimize cross-contamination between batches. A cotton ball saturated with a 10% sugar solution was placed on top of the screen for food.

The number of dead mosquitoes in each cup was recorded 24 h after dosing. The cup was then placed into a freezer for a sufficient time to immobilize the remaining live mosquitoes. Each replicate batch of mosquitoes were then transferred to a tared vial (one vial for each replicate batch), weighed to determine total mosquito mass in the sample and placed into a freezer for later analysis of permethrin in the sample. The total mass was divided by the number of mosquitoes in the sample to determine an average mosquito mass. In total, 15 vials of mosquitoes (5 dose levels and 3 toxicity tests per species) for both species were analyzed to determine permethrin dosed to the mosquitoes.

As an accuracy check of the intended dose to each mosquito, 200 nL (the same volume administered to each mosquito) of the mid-level dosing solution was pipetted into 1 mL of acetone in each of three amber vials (2-mL autosampler vials) and analyzed for permethrin. The expected permethrin mass in 200 nL of the mid-level dosing solution was 0.8 ng. The mass measured in the vials averaged 0.78 ng (0.67–0.82), which was 97% of nominal.

### Exposure Studies

Permethrin residues on adult butterflies and adult mosquitoes were quantified after their exposure to ULV sprays conducted in July–August of 2020 (Spray Set 1 or SS1) and from January to June of 2022 (Spray Set 2 or SS2). The first set of sprays (SS1) were conducted to evaluate the relation between permethrin exposure for butterflies and distance from the spray truck. The second set of sprays (SS2) were conducted to evaluate the influence of butterfly movement on their exposure to permethrin.

The mosquito species used in these studies (*Aedes albopictus*) was from a culture maintained by the City of Gainesville Mosquito Control. The age of this mosquito at the time of the field sprays was between 3 and 5 days. The butterfly species used for the sprays (*Dryas julia*) was obtained from Shady Oak Butterfly Farm, Brooker, Florida.

For both SS1 and SS2, between 10 and 27 live adult mosquitoes of the species *Aedes albopictus* (Asian tiger mosquito) and 5–10 live adult butterflies of the species *Dryas julia* (julia butterfly) were placed into separate cages at the laboratory, placed into clean coolers and then transported to the test site in the field approximately 20 min from the laboratory. The cages were constructed of open-ended cardboard cylinders approximately 18 cm in diameter and in length with fine mesh netting on either end to confine the butterflies and mosquitoes. Once prevailing wind direction was determined, the truck route and cage locations were marked such that the truck would drive perpendicular to wind direction and the cages would be downwind from the truck. After the cage locations were marked for the spray, the cages were removed from the coolers and placed at the respective locations approximately 10 min prior to the spray. The cages were secured to a polyvinyl chloride frame such that they were suspended approximately 1 m above the ground with the cage opening facing into the prevailing wind. The deployed cages were retrieved no sooner than 5 min after spray completion and returned to the same coolers for transport back to the laboratory.

Also for both SS1 and SS2, Aqua-Kontrol 30–30 (30% permethrin and 30% piperonyl butoxide) was diluted 1:5 (Aqua-Kontrol:water) and applied by a truck-mounted London Fogger ULV sprayer (London Foggers, Minneapolis, MN) at a rate of 0.00175 pounds active ingredient per acre with a target flow rate of 4.8 fluid ounces per min at a truck speed of 15 miles per hour. The flow rate was automatically adjusted for truck speed to maintain the application rate. All sprays occurred in an open field to enable flexibility for arrangement of cage locations in relation to prevailing wind direction and for setting the spray truck route.

In SS1, four sprays were conducted to evaluate exposure at different distances from the spray truck. For these sprays, paired cages of live butterflies and live mosquitoes were placed approximately 15, 45 and 75 m downwind of the spray truck as well as 30 m upwind. These sprays were conducted approximately 30 min after dusk when most of the live butterflies should be calm (roosting) mimicking the likely activity level for adult butterflies during the time of ULV sprays (after sunset). Prevailing wind speed ranged from calm to 6 mph (2.68 m/sec), while temperature and relative humidity ranged from 24.4 to 27.8 °C and 79 to 97%, respectively. Upon return to the laboratory, the butterflies and mosquitoes were transferred into clean cages, given a 10% sugar solution for food and monitored for 24 h. The number of dead and live butterflies and mosquitoes was noted at the end of 24 h. Then, all (live and dead) butterflies at each distance from the spray (upwind and downwind) were composited within pre-weighed containers, reweighed and placed into a freezer (− 20 °C) for later analysis of permethrin concentrations. The same was done with the mosquitoes. This resulted in 16 composite samples (one for each of four distances on four spray dates) for butterflies and for mosquitoes following these sprays. The composite mass for mosquitoes in each container was divided by the number of mosquitoes in the container to determine the average mosquito mass.

Ten sprays were conducted for SS2. For these sprays, a third cage containing 5 dead (i.e., motionless) julia butterflies were deployed alongside the cages containing live butterflies and mosquitoes. The dead butterflies were obtained by placing live butterflies into a freezer the night before the spray and then removing them the following morning. They were permitted to thaw in the laboratory before an insect pin was inserted through the thorax (dorsally to ventrally). Each dead butterfly was then pinned into a Styrofoam block affixed to the interior of the cage to ensure the butterflies were in an upright position during the sprays. Caged butterflies and mosquitoes were deployed approximately 30 m downwind of the spray truck. In contrast to the sprays evaluating the effect of distance, these sprays were conducted during the day to ensure there was wind to carry the aerosol cloud toward the cages. Prevailing wind speed ranged from 3 to 12 mph (1.34–5.36 m/sec) while temperature and relative humidity ranged from 24.4 to 33.3 °C and 24 to 54%, respectively. Condition of the mosquitoes and butterflies (alive, experiencing toxicosis, dead) was noted after returning them to the laboratory. Toxicosis was characterized as abnormal behavior (laying on side and convulsing or tremors for butterflies; frequent preening or leg cleaning for mosquitoes). After returning to the laboratory following a spray, all butterflies deployed as live were composited into pre-weighed containers, reweighed and frozen for analysis of permethrin residues. The same was done for the mosquitoes and the butterflies deployed as dead. This resulted in 10 composite samples each for live butterflies, dead butterflies and mosquitoes. Average mosquito mass was determined as described earlier. Between 45 and 60 min typically elapsed between completion of each spray and these samples being placed into the freezer.

### Residue Analyses

Frozen butterflies were freeze-dried and homogenized by Geno/Grinder® (SPEX SamplePrep) after which a portion (target of 0.2 g dry weight) was taken for extraction. Mosquito samples were air-dried in the dark until there was no mass change between consecutive measurements. Mosquitoes were air-dried rather than freeze-dried to minimize sample loss. Both matrices were spiked with 50 µL of 1 ng/µL surrogate solution of ^13^C_6_-*cis*-permethrin and extracted. The butterfly samples were extracted using an EDGE® extraction system (CEM Corporation) with 50:50 (v:v) hexane:acetone at 100 °C (hold time 3 min; total volume 30 mL), exchanged into dichloromethane (0.5 mL), loaded onto carbon solid-phase extraction cartridges (Restek CarboPrep 90; 6 mL, 500 mg) and eluted with 10 mL of dichloromethane. Mosquito samples were extracted by sonication for 10 min each in consecutive 5 mL volumes of dichloromethane that were combined after sonication. The dichloromethane eluent from the butterfly extracts and from the mosquito sample extracts was evaporated under nitrogen and exchanged into 0.2 mL acetonitrile to which was added 20 µL of a 2.5 ng/µL internal standard (bifenthrin-d5). The samples were filtered (0.45 µm PTFE) prior to analysis for (cis and trans) permethrin by gas chromatography–tandem mass spectrometry (GC-MS/MS; Gross et al 2021). Recovery of ^13^C_6_-*cis*-permethrin averaged 91.9% (95% CI of 70.4–109.5%). Concentrations reported in this manuscript were not corrected for permethrin recovery.

### Data Analysis

For each toxicity test with mosquitoes, response data were corrected for mortality in the solvent controls based on Abbott’s formula (Abbott 1925), converted to probits (Finney and Stevens 1948) and plotted in Microsoft Excel relative to the log of the permethrin concentration measured on the mosquitoes. The LD50 was estimated from the regression equation as the concentration (ng permethrin per gram of mosquito) equivalent to a probit of 5.

A 24-h LD50 was estimated for butterflies exposed during SS1. Permethrin concentrations on the butterflies after the spray were used as the exposure relative to response. As was done for the mosquitoes, a regression equation between log of the concentration and the probit was used to estimate the LD50.

Permethrin concentrations on the mosquitoes and butterflies following SS1 were compared (Kruskal–Wallis Test, *α* = 0.05) to determine significant differences among the distances from the spray truck. Permethrin concentrations on live and dead butterflies following SS2 were used to evaluate the influence of movement on butterfly exposure. The ratio of permethrin concentration on live and dead butterflies (live/dead) was calculated for each spray. Movement was presumed to have an influence on exposure if the lower limit of the 95% confidence interval for the average ratio was greater than one.

Relative exposure of colocated butterflies and mosquitoes to permethrin was evaluated for data from both SS1 and SS2. A ratio was calculated between permethrin concentrations on butterflies (deployed live and dead) relative to colocated mosquitoes (mosquito/butterfly) for each spray. Given the assumption that residues on dead butterflies represent roosting butterflies, the ratio between mosquitoes and dead butterflies may represent relative exposure in the field for roosting butterflies. Exposure of butterflies and mosquitoes was considered different if the lower limit of the 95% confidence interval around the average ratio was greater than one.

### Risk Estimation

Permethrin risk to adult butterflies and mosquitoes following ULV sprays was evaluated by comparing cumulative frequency distributions of measured exposures and available toxicity data. Toxicity data for five different mosquito species (Aa, Cq, *Aedes albopictus*, *Anopheles gambiae* and *Anopheles quadrimaculatus*) include those reported in the literature (Pridgeon et al. 2008; Chansang et al. 2018; Estep et al. 2018; Baker et al. 2023) and determined in the present study. Toxicity data for adult butterflies (*Heliconius charitonia*, *Eumaeus atala*, *Junonia coenia*, *Vanessa cardui*, *Anartia jatrophae*) also include those reported in the literature (Salvato 2001; Hoang et al. 2011). When multiple toxicity values (24-h LD50s) for a single species were available, the median of the values was used in the frequency distribution rather than all the available toxicity values to avoid weighting the distribution toward the sensitivity of a single species. The exposures used for the risk estimation were those measured (permethrin mass per unit mass of butterflies or mosquitoes). The latter was estimated from the measured concentration in composited mosquito samples along with the mass of the respective composite sample and number of mosquitoes in the sample (ng/mosquito = [concentration (ng/g) × mass (g)] ÷ number of mosquitoes). Risk was presumed if the measured or estimated exposures exceeded the toxicity values. Relative risk between butterflies and mosquitoes was evaluated by the magnitude by which exposures exceeded toxicity.

## Results

All data associated with this manuscript are publicly available in Bargar (2023).

### Permethrin Toxicity

**Fig. 2:**
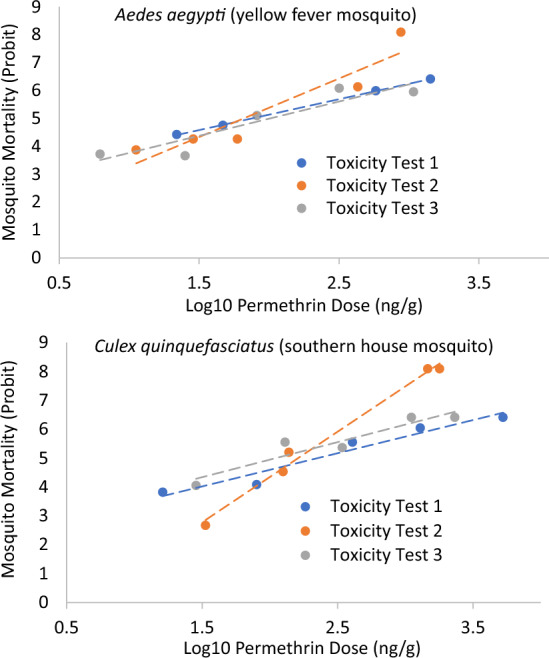
Permethrin concentrations measured on adult mosquitoes in relation to mortality for those mosquitoes in three separate toxicity tests for *Aedes aegypti* and *Culex quinquefasciatus*

Figure 2 shows results of the toxicity tests with adult Aa and Cq. The estimated 24-h LD50 values based on measured permethrin exposure were 81.1 ng/g dw (75.5–101.9) for Aa and 166.3 ng/g dw (110.7–226.3) for Cq.

The permethrin mass per mosquito equivalent to the LD50 was estimated from the product of the LD50 (ng/g) and individual mosquito mass (g). The total of number of Aa mosquitoes among the three toxicity tests was 197 with a mass of 0.3622 g resulting in an average mosquito mass of 1.8 mg. The total of number of Cq mosquitoes among the three toxicity tests was 213 with a mass of 0.4987 g resulting in an average mass of 2.3 mg. Based on those individual mosquito masses and the LD50 values, the permethrin mass per mosquito equivalent to the LD50 was 0.16 and 0.4 ng for Aa and Cq, respectively.

**Fig. 3:**
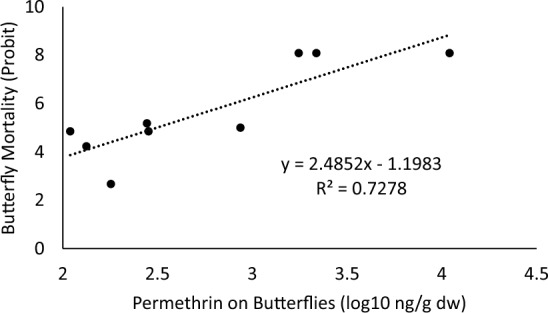
Relation between permethrin concentrations on julia butterflies (*Dryas Julia*) and their mortality following their exposure to ultra-low-volume sprays of a pesticide containing permethrin

Figure 3 shows the relation between butterfly mortality and the measured exposure following the ULV sprays. The estimated LD50 based on that relation was 312 ng/g. It should be noted that, in contrast to the toxicity values noted in Hoang et al. (2011) based on permethrin alone, this toxicity value is based on butterfly exposure to Aqua-Kontrol 30–30, which is a formulation containing both the active ingredient permethrin and the synergist piperonyl butoxide.

Mosquito mortality following the sprays ranged from 53 to 100% when response was assessed at 1 h post-spray and from 80 to 100% when assessed at 24 h post-spray, indicating most mosquitoes died rapidly following exposure. Butterfly mortality following the sprays ranged from 0 to 17% at 1 h post-spray and from 0 to 100% at 24 h post-spray, indicating butterfly response was delayed relative to mosquito response; however, most of the butterflies alive at 1 h post-spray exhibited signs of toxicosis. No mosquito or butterfly mortality occurred at the upwind locations.

### Exposure Following ULV Sprays

Permethrin concentrations (ng/g dw) on mosquitoes (from SS1 and SS2) and on butterflies deployed live (SS1 and SS2) ranged from 911.7 to 38,060.6 and from 109.6 to 11,004, respectively. Concentrations on butterflies deployed dead (SS2 only) ranged from 113.1 to 2224.5. No permethrin was detected in butterfly and mosquito samples from cages deployed upwind of the spray truck.

**Fig. 4:**
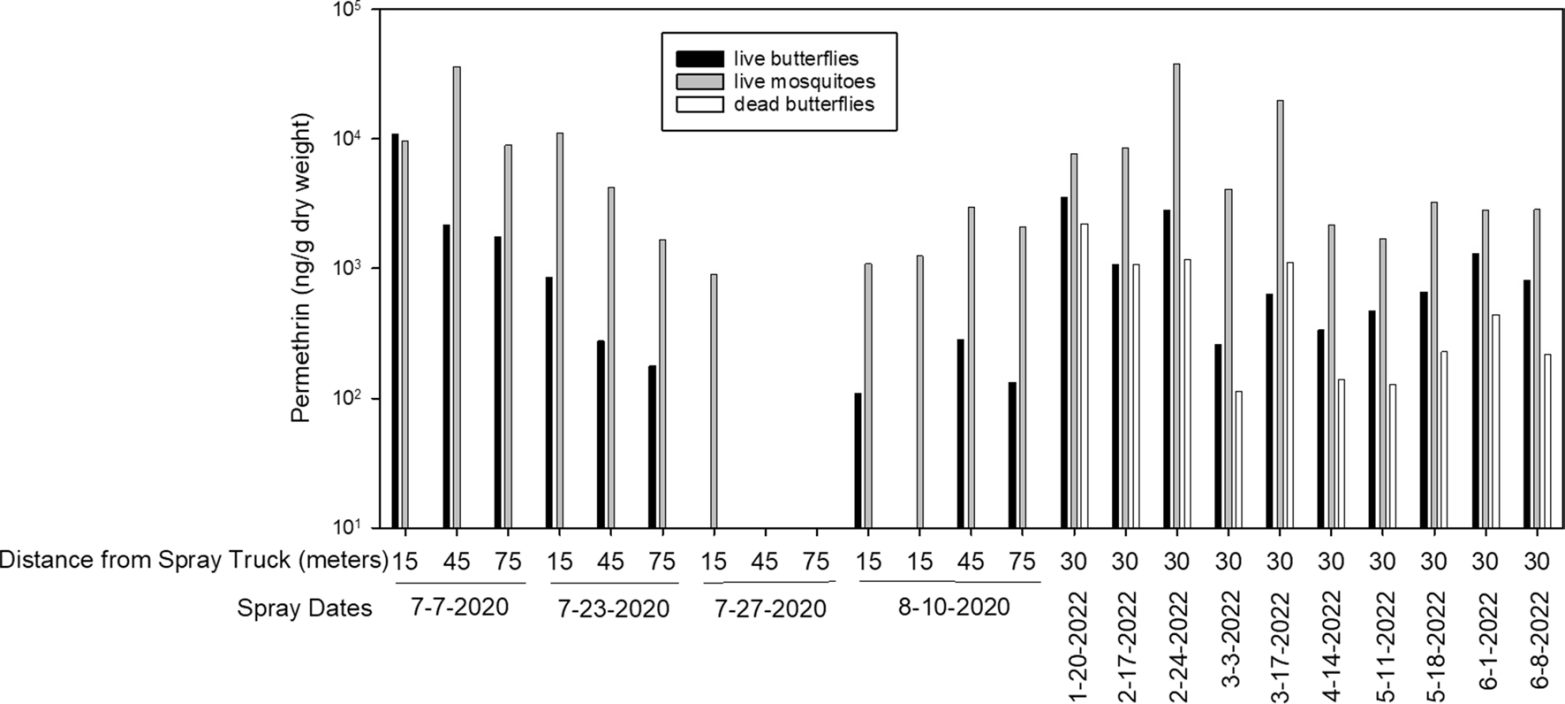
Measured permethrin concentrations on adult mosquitoes (*Aedes albopictus*) in relation to concentrations on colocated adult julia butterflies (*Dryas julia*) following each of 14 ultra-low-volume sprays. Reference in the legend to live and dead butterflies and to live mosquitoes describes their condition when they were deployed prior to, not after, the sprays

While the concentration ranges for the three groups (mosquitoes, live and dead butterflies) overlap among the sprays, concentrations on colocated insects typically differed within a spray date (Fig. 4). Concentrations on the live butterflies on average were 2.3 times greater (95% CI 1.6–3.1) than on the colocated dead butterflies, indicating that movement may enhance butterfly exposure to permethrin in a ULV spray. Except for one instance (15 m from spray truck for the spray on July 7, 2020), permethrin concentrations on average were 9.9 times higher (95% CI 6.4–13.4) on mosquitoes than on colocated live butterflies. Relative to dead butterflies, permethrin concentrations were 16.1 times higher (95% CI 8.5–23.7) on mosquitoes.

Individual mosquito exposure to permethrin (ng per mosquito) during the ULV sprays was estimated based on the measured concentrations, the number of mosquitoes in the composite samples and the total mass of the samples (Table 1). The average for all sprays ranged from 0.3 to 17.1 ng/mosquito.

**Table 1:** Average permethrin mass per mosquito following exposure to ultra-low-volume sprays

Spray date	Distance from truck (m)	Permethrin concentration (ng/g dw)	Sample mass (g dw)^a^	Permethrin in sample^b^ (ng)	Number of mosquitoes	Average ng permethrin per mosquito^c^
7-7-2020	30 upwind	0	0.0148	0	10	0
7-7-2020	15	9785.5	0.0098	95.9	10	9.6
7-7-2020	46	36,247.8	0.0028	101.5	10	10.1
7-7-2020	76	9024.2	0.0040	36.1	9	4.0
7-23-2020	30 upwind	0	0.0194	0	21	0
7-23-2020	15	11,115.0	0.0100	111.1	27	4.1
7-23-2020	46	4261.7	0.0086	36.7	26	1.4
7-23-2020	76	1688.0	0.0090	15.2	26	0.6
7-27-2020	30 upwind	0	0.0647	0	22	0
7-27-2020	15	911.7	0.0075	6.8	20	0.3
8-10-2020	30 upwind	0	0.0424	0	24	0
8-10-2020	15	1093.9	0.0060	6.6	25	0.3
8-10-2020	15	1259.7	0.0106	13.4	25	0.5
8-10-2020	46	2990.4	0.0099	29.6	22	1.3
8-10-2020	76	2120.3	0.0107	22.7	25	0.9
1-20-2022	30	7721.5	0.0079	61.0	23	2.7
2-17-2022	30	8612.9	0.0124	106.8	25	4.3
2-24-2022	30	38,060.6	0.0099	376.8	22	17.1
3-3-2022	30	4113.6	0.0088	36.2	15	2.4
3-17-2022	30	19,978.5	0.0093	185.8	24	7.7
4-14-2022	30	2200.0	0.0110	24.2	24	1.0
5-11-2022	30	1717.0	0.0212	36.4	25	1.5
5-18-2022	30	3257.9	0.0159	51.8	17	3.0
6-1-2022	30	2842.1	0.0038	10.8	17	0.6
6-8-2022	30	2874.4	0.0199	57.2	26	2.2

^a^mosquito mass for samples 30 m upwind are fresh weight

^b^permethrin in sample (ng) = permethrin concentration (ng/g) × sample mass (g)

^c^ng per mosquito = permethrin in sample ÷ number of mosquitoes

**Fig. 5:**
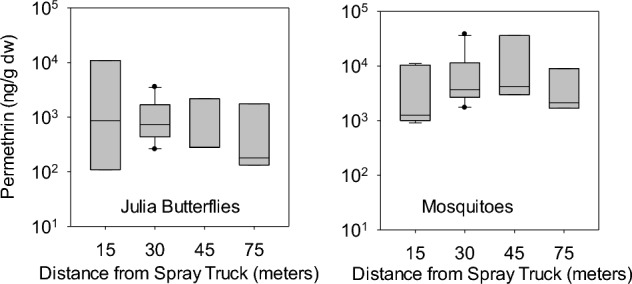
Relation between the distance from the spray truck and permethrin concentrations measured on adult julia butterflies (*Dryas julia*) and adult mosquitoes (*Aedes albopictus*). Differences among distances were not significant (Kruskal–Wallis Test) for either mosquitoes (*p* = 0.45) or butterflies (*p* = 0.67)

While concentrations on live butterflies appeared to decline with distance from the spray truck (Fig. 5), they were not significantly different (*p* = *0.67*). Concentrations on mosquitoes were higher at 30 and 45 m from the spray truck relative to 15 and 75 m, but also were not significantly different (*p* = *0.45*).

### Risk Estimation

**Fig. 6:**
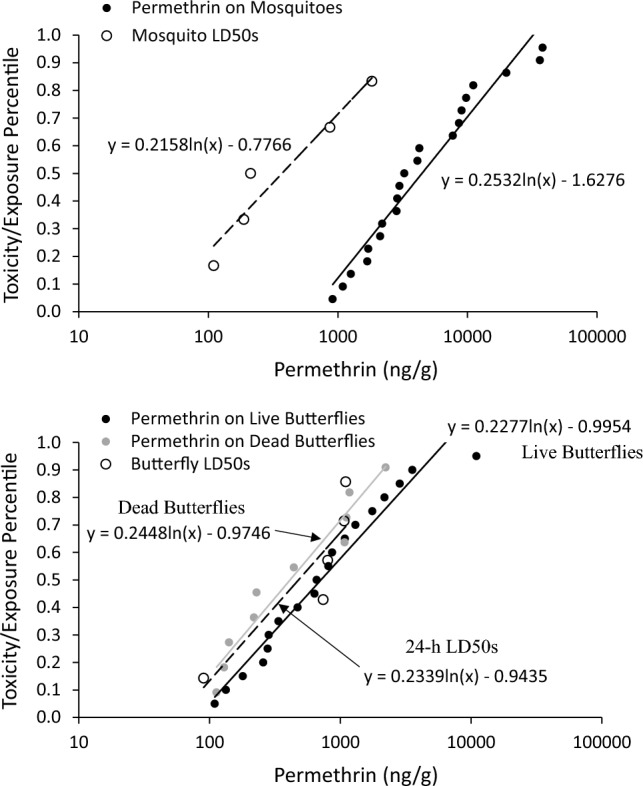
Frequency distributions of permethrin concentrations measured on adult mosquitoes (top panel) and on adult butterflies (bottom panel) following their exposure to an ultra-low-volume spray containing permethrin in relation to species sensitivity distributions for permethrin acute toxicity (24-h LD50) to adult mosquitoes and adult butterflies. Equations are for the best-fit logarithmic regressions for the respective exposure or toxicity data. Reference in the legend to live and dead butterflies reflects their condition when they were deployed prior to, not after, the sprays

Cumulative frequency distributions for the toxicity and measured exposure data are shown in Fig. 6. The distributions for mosquitoes. The range of median 24-h LD50s for mosquitoes (110–1830 ng/g) was lower than the range of permethrin residues measured on the mosquitoes following the ULV sprays (912–38,060 ng/g). The 50th percentile of the measured exposures (4,489 ng/g) was approximately 12-fold greater than the 50th percentile of the 24-h LD50s (371 ng/g). The lowest measured exposure (912 ng/g) was equivalent to the 69th percentile of the toxicity distribution. Both indicate a high level of risk for mosquitoes following the ULV sprays.

Relative to data for the mosquitoes, the range of median 24-h LD50 values for butterflies (90–1100 ng/g) was similar to the range of permethrin residues measured following the ULV sprays (110–11,004 ng/g) (Fig. 6). An indication of that similarity is the ratio between the 50th percentile exposure values and the 50th percentile of the toxicity distribution (479 ng/g). The 50th percentile exposures for butterflies deployed live and deployed dead were 712 and 413 ng/g, respectively. The exposure for butterflies deployed live was 1.5-fold greater than the 50th percentile toxicity value. Exposure for butterflies deployed dead was a little less than the 50th percentile toxicity value (ratio = 0.9). Both ratios are lower than those determined for mosquitoes, indicating lower relative risk for butterflies. While these data indicate lower relative risk for butterflies, their exposure to permethrin during a few of the ULV sprays was still adequate to result in 100% mortality.

All estimates of individual mosquito exposure after the sprays (0.3–17.1 ng/mosquito—Table 1) exceeded the exposure equivalent to the LD50 for Aa (0.16 ng/mosquito), while 19 of the 21 estimated exposures exceeded the exposure equivalent to the LD50 for Cq (0.4 ng/mosquito). The ratio between the estimated ng/mosquito following the sprays and the ng/mosquito equivalent to the LD50s for Aa and Cq ranged from 1.87 to 106.9 and 0.7 to 42.7, respectively, indicating high likelihood of mosquito mortality following the sprays.

## Discussion

Permethrin is a broad-spectrum insecticide that affects the nervous system of targeted insects (USEPA 2007). Data from toxicity studies submitted to the USEPA during the registration and reregistration process for permethrin indicate it is highly toxic to honeybees and beneficial insects but practically nontoxic (acute exposures) to mammals and birds (USEPA 2007). That differential sensitivity between the target insects and nontarget vertebrate organisms makes permethrin a desirable insect pest control tool. In fact, permethrin has been incorporated within articles of clothing and other materials to reduce interaction with disease vectors like mosquitoes and ticks (Banks et al. 2015; Prose et al. 2018; Panthawong et al. 2020). However, conservation of nontarget invertebrates such as imperiled butterflies is problematic when they cohabitate with targeted insects like mosquitoes. The present study was conducted to evaluate the relative risk for imperiled butterflies and mosquitoes to help resource managers responsible for their conservation understand the risk for the butterflies.

Permethrin toxicity to mosquitoes has been well investigated given its use for the control of adult mosquitoes (Zhang et al. 2015; Whiten and Peterson 2016; Gong et al. 2017; Sanchez-Arroyo et al. 2021; 2022), but the concentrations affecting the mosquitoes are often reported as nominal. Assumptions about exposures based on dose solution concentrations or possible unintended errors in toxicant delivery to an organism the size of an adult mosquito could lead to inaccuracies in the reported effect concentrations. Since interpreting residues measured on mosquitoes following ULV sprays was a goal of the present study, we analyzed permethrin concentrations on the mosquitoes following the toxicity tests to minimize potential errors in the reported effect concentrations. The 24-h LD50 we determined for Aa (81 ng/g) was within the range of toxicity values (17–440 ng/g) determined for Aa strains reported as susceptible to permethrin (Agramonte et al. 2017; Chansang et al. 2018; Estep et al. 2018; Tak et al. 2020). It was lower than the toxicity values for strains reported as relatively resistant to permethrin (350–6690 ng/g) (Estep et al. 2018). This indicates the Aa strain used in this study is more susceptible to permethrin than strains of wild Aa populations likely exposed to permethrin from mosquito control sprays and that the toxicity value determined in this study may overestimate sensitivity of wild Aa populations. The 24-h LD50 value determined in this study (166.3 ng/g) for Cq was less than the value (1600 ng/g) reported in Pridgeon et al. (2008). This large difference could reflect a real sensitivity difference between the tested mosquitoes but could also be the result of the aforementioned assumptions about exposure. No other studies were found that reported 24-h LD50 values as concentrations in adult Cq. Li and Liu (2010) reported toxicity values for Cq as permethrin mass per mosquito, but did not report the mass of those mosquitoes. The mass of Cq in the toxicity studies in this study was approximately 3 mg. Converting the toxicity values in Li and Liu (2010) to concentrations utilizing the mass from this study yielded estimated 24-h LD50s ranging from 62 to 625 ng/g for a permethrin-susceptible strain and from 94 to 62,500 ng/g for strains that had developed some resistance. The toxicity value for Cq determined in this study may also overestimate the sensitivity of the mosquito species to permethrin. It should be noted that some pesticide products containing permethrin as the active ingredient also contain the synergist piperonyl butoxide, which decreases the activity of enzymes responsible for metabolic degradation of permethrin thereby enhancing its toxicity (Amweg et al. 2006) and compensating for developed resistance. Development of resistance in natural adult mosquito populations complicates characterization of their sensitivity to permethrin in relation to the sensitivity of adult butterflies.

Few studies reporting permethrin toxicity to adult butterflies have been published, possibly because they are not standard toxicity test organisms and are not a targeted pest species. The possible risk of permethrin to imperiled butterflies in the Florida Keys was the impetus for the two studies that do report permethrin toxicity to butterflies (Salvato 2001; Hoang et al. 2011). The range of 24-h LD50s reported in those studies for five different butterfly species was 0.4–1600 ng/g. Relative to the range of 24-h LD50s reported for adult mosquitoes, there is an overlap of sensitivities for both insect groups exposed to permethrin. The higher toxicity values in the range for mosquitoes are the result of resistance development in wild Aa populations in Florida (Estep et al. 2018). Interestingly, the same study found little resistance development in colocated *Aedes albopictus* despite presumably similar exposures to permethrin, meaning resistance development to insecticides is not consistent among mosquito species. It is possible that nontarget Lepidopterans could also develop resistance to permethrin. Resistance development in pest Lepidopteran species has been reported (Jia et al. 2009; Grigg-McGuffin et al. 2015; Uesugi 2020; Sun et al. 2022) and has been reported in a nontarget aquatic invertebrate (Major et al. 2022). The common aspect of this resistance development is that it occurs in organisms either targeted by the applications or are unintentionally routinely exposed to the insecticide. At this point, no studies report resistance development in nontarget butterflies unintentionally exposed to permethrin from mosquito control sprays. Resistance of wild mosquito populations to permethrin has been reported (Estep et al. 2018; Li and Liu 2010). Such resistance development could lead to a shift of relative sensitivity of adult mosquitoes and adult butterflies impacting relative risk and the likelihood that one group could be preferentially affected by a spray.

To our knowledge, this study is the first to report permethrin residues on adult mosquitoes and butterflies following exposure to a ULV spray in the field. Zhang et al. (2015) exposed adult mosquitoes in an exposure chamber and estimated pyrethrin insecticide (phenothrin, bioresmethrin) concentrations on mosquitoes based on dye rinsed from the mosquitoes and average mosquito mass. Permethrin concentrations on adult mosquitoes following the ULV sprays in the present study were, on average, nearly an order of magnitude greater than they were on colocated live butterflies and even greater relative to dead butterflies. This indicates that, given the apparent similarity of adult butterfly and mosquito sensitivity to permethrin, a ULV spray will result in greater risk for adult mosquitoes than for adult butterflies. However, since ULV sprays for control of adult mosquitoes occur late in the evening when butterflies are roosting and relatively motionless, exposure of relatively motionless dead butterflies was also measured in this study. Published studies have shown that flight enhances mosquito exposure to insecticides in ULV sprays (David 1945; David and Bracey 1946), which may also be true for adult butterflies. Data from the present study indicate that exposure of live butterflies (moving) was greater relative to dead (relatively motionless) butterflies, and exposure of both was lower relative to mosquitoes.

This study attempted to evaluate how butterfly movement and distance from the sprayer may affect exposure. Prior studies with mosquitoes have shown that flight (movement) enhances their exposure to insecticides in ULV sprays and that the lack of movement minimizes effect and concordantly exposure (David and Bracey 1946). It is reasonable to assume butterfly exposure would also be enhanced by movement, but no prior studies have investigated adult butterfly exposure to insecticides in ULV sprays or if movement might increase their exposure. This study found that permethrin residues on dead butterflies were lower than on live butterflies, but not significantly lower. Air movement (i.e., wind) could enhance exposure of motionless (i.e., roosting) butterflies to the smaller droplets in a ULV spray. David and Bracey (1946) reported that mortality of motionless mosquitoes (immobilized by exposure to chloroform) increased from 10 to 78% as wind increased from 0.5 to 3.0 mph (0.22–1.34 m/sec). Himel and Moore (1967) collected spruce budworm larvae (motionless relative to flying mosquitoes) that had succumbed to an aerial application of a carbamate insecticide and analyzed the larvae to determine the droplet sizes on the larvae. They reported that the diameter of 92% of the droplets on the larvae were smaller than 30 µm. Both studies indicate that exposure to droplets in the size range of ULV sprays is not entirely dependent on insect movement when there is air movement. We found permethrin residues on dead (i.e., motionless) butterflies under the conditions of this study, most likely because wind speeds were sufficient to give the droplets adequate inertia to deposit onto the dead butterflies. Perhaps little to no permethrin would have deposited onto the dead butterflies under calm to near calm conditions. However, since some air movement is necessary to carry the spray cloud into areas targeted by a ULV spray (Mount 1998), exposure of roosting and motionless insects is likely.

Permethrin concentrations on butterflies and mosquitoes changed little with distance from the spray truck. The distance a spray droplet will travel can be estimated based on wind speed and particle settling velocity as calculated by Stoke's law (Bonds 2012). Under the same wind speed, the distance a particle or spray droplet will travel before settling to the ground is proportional to its diameter. Bonds (2012) estimated a 10 µm droplet, a size in the middle of sizes that deposit onto flying mosquitoes (Lofgren et al. 1973), would travel 144 m at a wind speed of 0.44 m/sec (~ 1 mph) and 575 m at a wind speed of 1.78 m/sec (~ 4 mph). Since the label for the product used in the present study (Aqua-Kontrol 30–30) indicates applications in wind speeds up to 10 mph (4.47 m/sec) are permitted, a 10 µm droplet could travel much further than the distances from the spray truck investigated in the present study. In this study, the greatest distance from the spray truck was 75 m and the wind speed ranged up to 5.36 m/sec. It is likely that only the largest droplets would have settled to the ground within 75 m of the spray truck, meaning the droplet distribution of the spray cloud at each of the tested locations would have differed little. Correspondingly, differences of permethrin residues on butterflies and mosquitoes would be unlikely among the locations.

Adult mosquito exposure to permethrin following the ULV sprays greatly exceeded that necessary to result in their mortality. The excess exposure was likely due to the cage deployment location. The caged mosquitoes were deployed in an open field where, because of the lack of obstructions that could intercept spray droplets, exposure would be relatively high compared to exposure in a habitat with greater vegetation density. The effect of vegetation on mosquito exposure was indicated by data reported in Rathburn and Dukes (1989) and in Jiang et al. (2020). Droplet samplers they deployed in open habitat collected a larger volume of droplets (Rathburn and Dukes 1989) or a greater mass of permethrin (Jiang et al. 2020) relative to samplers in a more densely vegetated habitat. Both Rathburn and Dukes (1989) and Barber et al. (2007) reported that adult mosquito mortality following a ULV spray was higher in open habitat relative to more densely vegetated habitat. This moderating effect of vegetation on spray efficacy against mosquitoes is likely the reason for increased application rates needed to attain adequate efficacy against mosquitoes in more densely vegetated locations (Mount et al. 1996; Rathburn and Dukes 1989). It is likely that the difference between the exposure and sensitivity distributions determined for mosquitoes in the present study would have been less if the exposures had been measured for mosquitoes in cages deployed in more densely vegetated habitat.

The difference between the exposure and sensitivity distributions for adult butterflies was small compared to that for mosquitoes. This likely was the result of lower activity levels compared to mosquitoes. Despite the lower exposure relative to toxicity, exposure was still sufficient to result in mortality up to 100% following some of the sprays. Placing the butterflies in a densely vegetated habitat may have reduced their exposure and risk, but the present study did not evaluate that scenario. Barber et al. (2007) reported that mosquito mortality in vegetated habitat was reduced 15–78% relative to mortality in open habitat. The magnitude of exposure and effect reduction that butterflies may experience in vegetated habitat is unknown, as is the significance of that possible reduction for butterfly conservation.

Results of this study indicate that permethrin in ULV sprays may present a lower risk for adult butterflies relative to adult mosquitoes. However, that lower risk did not translate to minimal effect because the exposure was enough to result in up to 100% mortality in the open-field environment. Under those conditions, the application rate would have to be reduced to minimize effects for butterflies, at least for the species used in this study. Lowering the application rate would likely reduce exposure for butterflies and mosquitoes. Based on data from this study, in an open field it may be possible to reduce the application to minimize effects for butterflies and still achieve elevated mosquito mortality. However, areas exposed to ULV sprays are often a mixture of open and densely vegetated habitat or residential areas where spray efficacy against mosquitoes is reduced (Rathburn and Dukes 1989; Barber et al. 2007; Jiang et al. 2020), which means elevated applications are necessary to reach desired efficacy (Mount et al. 1996). It is uncertain how representative results of this study would be for butterflies in habitats adjacent to residential areas routinely subjected to ULV applications. Exposure of butterflies in the field may be reduced if they are in densely vegetated habitats, but to our knowledge, that scenario has not been tested for ULV sprays from truck-mounted sprayers. In addition, it is uncertain how representative the julia butterflies might be of untested species given the lack of data on permethrin toxicity to adult butterflies. Toxicity testing with additional butterfly species may increase certainty in the risk estimated for imperiled butterfly species exposed to ULV sprays.

## Data Availability

The data on which the figures and analyses in this paper are based are publicly available in Bargar (2023) (https://doi.org/10.5066/P9QUE6DV).
